# Preparing Alumina-Supported Gold Nanowires for Alcohol Oxidation

**DOI:** 10.1021/acsomega.1c01895

**Published:** 2021-06-13

**Authors:** Yoshiro Imura, Motoki Maniwa, Kazuki Iida, Haruna Saito, Clara Morita-Imura, Takeshi Kawai

**Affiliations:** † Department of Industrial Chemistry, Tokyo University of Science, 1-3 Kagurazaka, Shinjuku-ku, Tokyo 162-8601, Japan; ‡ Department of Chemistry, Faculty of Science, Ochanomizu University, 2-1-1 Otsuka, Bunkyo-ku, Tokyo 112-8610, Japan

## Abstract

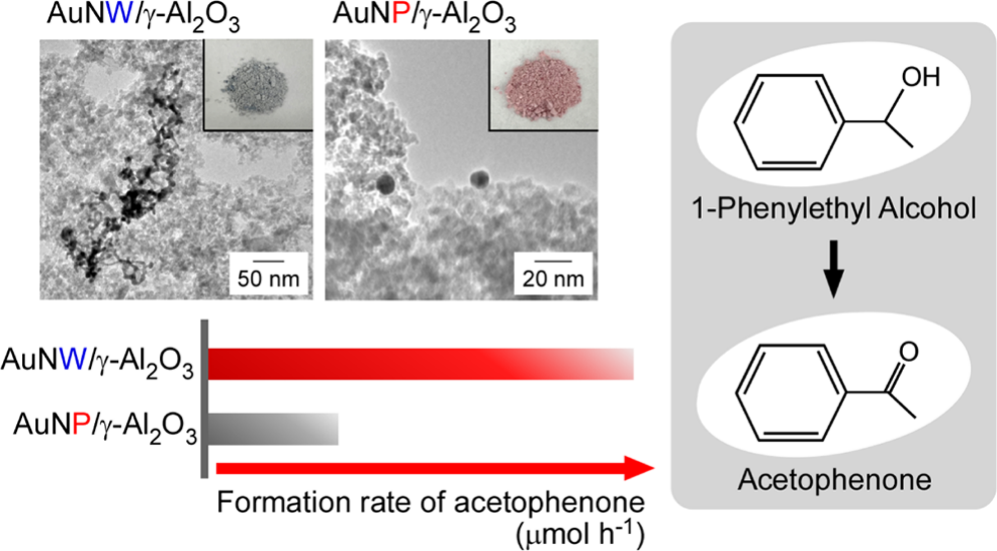

The development of shape-controlled noble metal nanocrystals such as nanowires (NWs) is progressing steadily owing to their potentially novel catalytic properties and the ease with which they can be prepared by reducing the metal ions in a particular solution as capping agents. Recently, many reports have been presented on the preparation of shape-controlled Au nanocrystals, such as nanostars and nanoflowers, by a one-pot method using 2-[4-(2-hydroxyethyl)-1-piperazinyl] ethanesulfonic acid (HEPES) as capping and reducing agents. The catalytic activity is depressed due to the adsorption of the capping agent onto a Au surface. Since HEPES has low binding affinities on the Au surface, shape-controlled nanocrystals obtained using HEPES are effective for application as nanocatalysts because HEPES was easily removed from the Au surface. In this study, we report the preparation of AuNWs, with an average diameter of 7.7 nm and lengths of a few hundred nanometers, in an aqueous solution containing HEPES and sodium borohydride. A γ-Al_2_O_3_-supported AuNW (AuNW/γ-Al_2_O_3_) catalyst was obtained using catalytic supporters and a water extraction method that removed HEPES from the Au surface without morphological changes. AuNW/γ-Al_2_O_3_ was then utilized to catalyze the oxidation of 1-phenylethyl alcohol to acetophenone. The formation rate of acetophenone over AuNW/γ-Al_2_O_3_ was 3.2 times that over γ-Al_2_O_3_-supported spherical Au nanoparticles (AuNP/γ-Al_2_O_3_) with almost the same diameter.

## 1 Introduction

**Figure 1:**
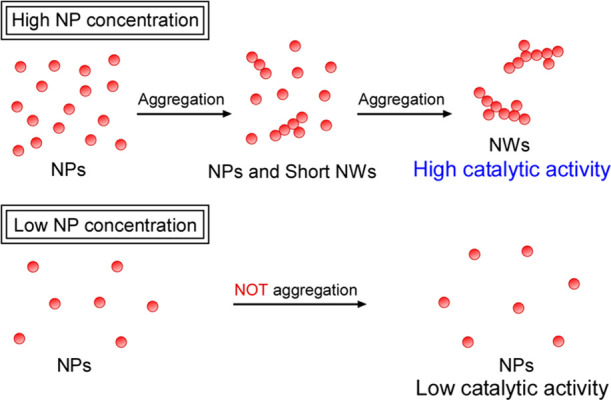
Schematic diagram illustrating the formation of AuNWs.

The study of noble metal nanocrystals is very important in several areas of nanosciences, such as electrochemistry, electronics, magnetic storage sensing, and catalysis.^1−10^ The properties of metal nanocrystals are strongly dependent on their sizes and shapes.^1,2,11^ Therefore, an effective synthesis technique is essential to obtain nanocrystals with desired properties. Shape-controlled nanocrystals, such as nanowires (NWs) and nanoflowers, are easily synthesized by reducing the noble metal ions in a solution containing a surfactant, a polymer, or low molecular organic components as capping agents, which inhibit the precipitation of nanocrystals.^12−22^ However, for gold nanoparticles (NPs), spherical NPs, such as decahedral, icosahedral, and truncated octahedral nanocrystals, are identified as stable structures because the surface area per volume of spherical NPs is lower than that of shape-controlled nanocrystals.^1,23^ Recently, many studies have reported that NWs are prepared using the diffusion-limited aggregation method.^1,24−27^ Such NWs tend to exhibit high catalytic activities compared to the original spherical NPs owing to the disordered state of the metal atoms within the aggregated domains, namely, grain boundaries, which often exhibit high catalytic activity.^25−30^ To prepare NWs by this method, it needs high NP concentration because of the difficulty to aggregate NPs under low concentration (Figure 1).

When shape-controlled nanocrystals are applied to a catalyst, the catalytic activity is depressed due to the adsorption of the capping agent onto the metal surface.^31−34^ Thus, it is important to remove the capping agent from the metal surface after the preparation of NWs. However, the capping agent cannot be completely removed without the precipitation of shape-controlled nanocrystals.^34^ Catalytic support materials such as γ-Al_2_O_3_, SiO_2_, and carbon support are known to improve the dispersion stabilities of nanocrystals.^34−40^ γ-Al_2_O_3_ shows relatively strong interaction with metal nanocrystals, while SiO_2_ and the carbon support have relatively weak metal–support interaction.^34,39,40^ For catalytic applications, the capping agent must be removed after supporting the shape-controlled nanocrystals.^34^ Hence, the use of a capping agent with weak adsorption properties is important as it can be easily removed from the metal surface via an extraction procedure.^34,35,41,42^

Extensive research has been conducted on the preparation of shape-controlled metal nanocrystals using 2-[4-(2-hydroxyethyl)-1-piperazinyl] ethanesulfonic acid (HEPES) (Figure S1) because it can be functionalized to other molecules because of its low binding affinities on metal surfaces.^43−54^ The weak adsorption property is effective for application as nanocatalysts. HEPES acts as both capping and reducing agents, and the shape-controlled nanocrystals can be easily obtained in a liquid phase.^43−54^ To date, many studies have described the preparation of nanoflowers,^43,44^ nanostars,^45−47^ tetrapod nanocrystals,^48,49^ multibranched nanocrystals,^50−52^ and spiky nanocrystals^53^ using HEPES as capping and reducing agents. We observed that the shape of the nanocrystals was affected by both these agents. The addition of a reducing agent such as sodium borohydride (NaBH_4_) is expected to prepare specific shape-controlled nanocrystals. In this study, we report the preparation of AuNWs obtained by reducing Au ions in aqueous HEPES and NaBH_4_ and its catalytic performance for the oxidation reaction of 1-phenylethyl alcohol to acetophenone.

## 2 Results and Discussion

**Figure 2:**
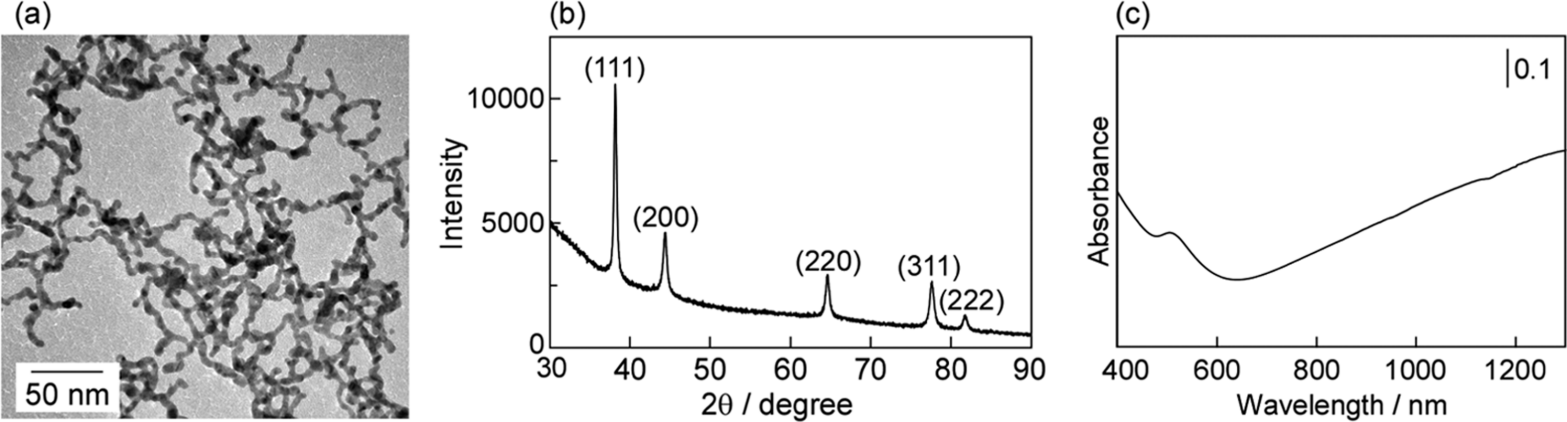
(a) TEM image, (b) X-ray diffraction pattern, and (c) UV–vis absorption spectrum of AuNWs. The concentrations of HEPES and NaBH_4_ are 15 and 110 mM, respectively.

Transmission electron microscopy (TEM) images showed that AuNWs with an average diameter of 7.7 nm were obtained by mixing an aqueous solution of NaBH_4_ (110 mM) with an aqueous solution containing HEPES (15 mM) and HAuCl_4_ (Figure 2a). TEM-energy-dispersive X-ray (EDX) revealed that the AuNWs were composed of pure Au (Figure S2). The X-ray diffraction (XRD) peaks of AuNWs at 38.2, 44.4, 64.6, 77.6, and 81.6° were assigned to the (111), (200), (220), (311), and (222) diffractions of face-centered cubic Au, respectively (Figure 2b).^55,56^ UV–vis spectroscopy is a useful tool for examining the shape of Au nanocrystals because the surface plasmon (SP) band of a Au nanocrystal is strongly dependent on its shape.^34,57−59^ The SP band of the NW structure is observed at high wavelengths such as the infrared region where absorption occurs (Figure 2c).^16,58^ In addition, AuNWs were also prepared using 50, 100, and 200 mM HEPES aqueous solutions containing NaBH_4_ (Figure S3).

**Figure 3:**
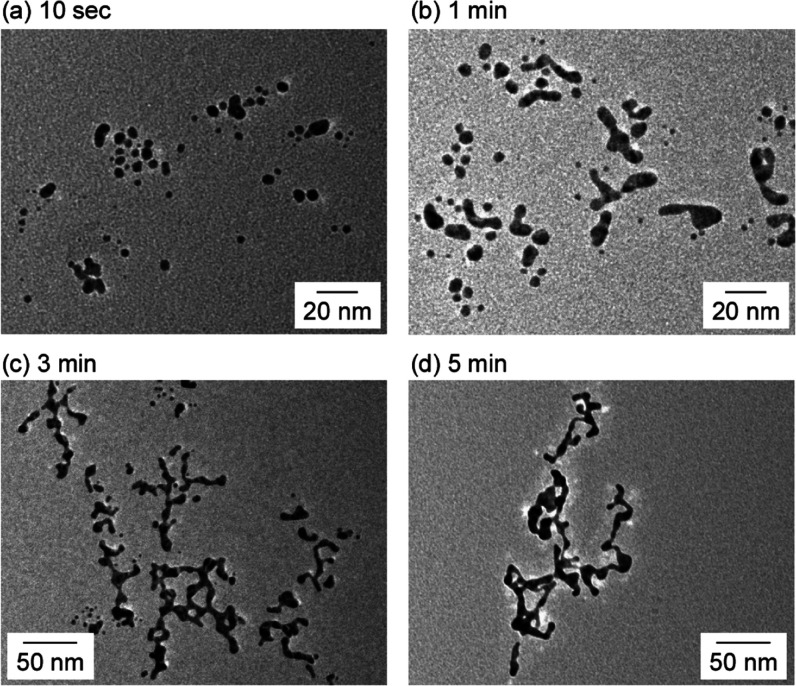
TEM images of Au nanocrystals at (a) 10 s, (b) 1 min, (c) 3 min, and (d) 5 min after the addition of NaBH_4_. The concentrations of HEPES and NaBH_4_ are 15 and 110 mM, respectively.

**Figure 4:**
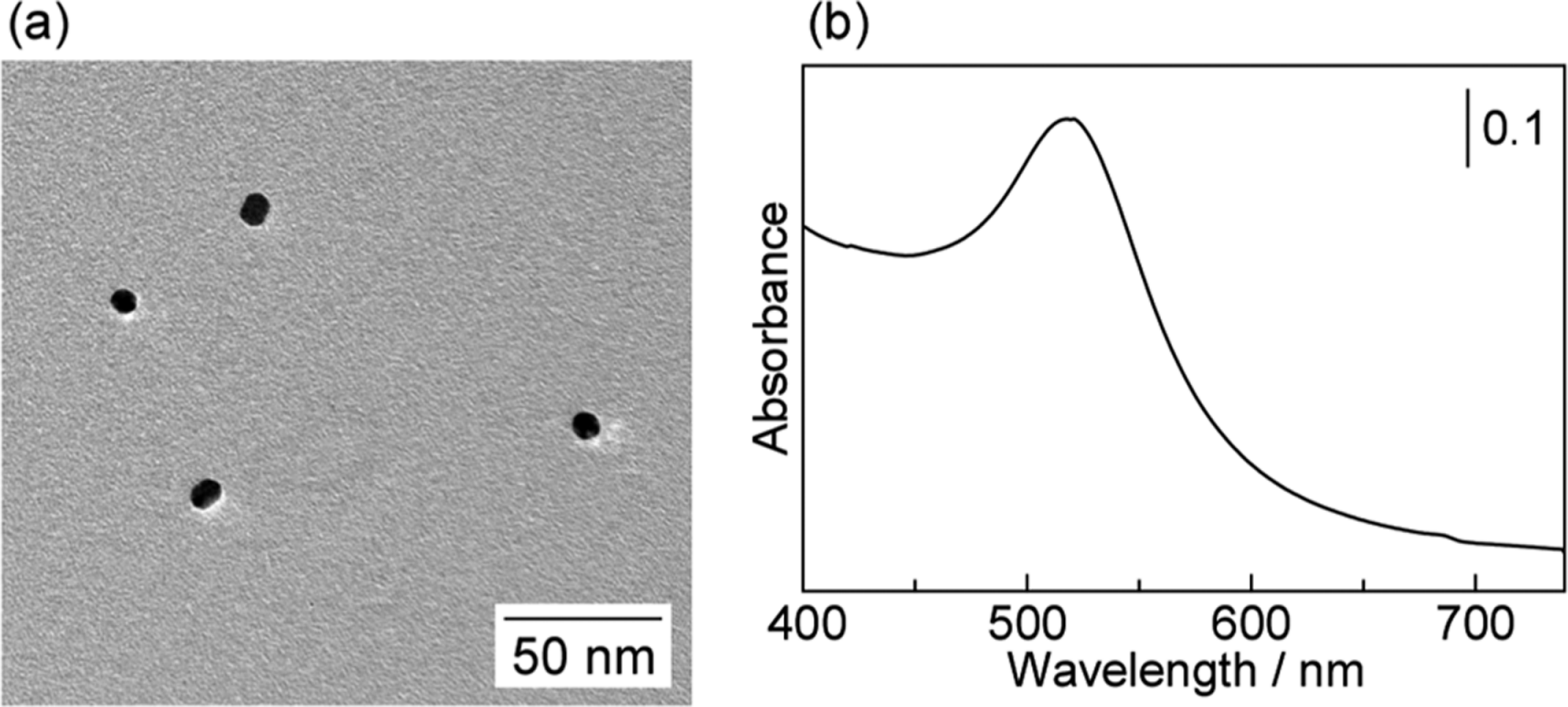
(a) TEM image and the (b) UV–vis spectrum of spherical AuNPs.

To examine the mechanism of formation of AuNWs, TEM measurements were performed at various reaction times (Figure 3). After 10 s, only spherical AuNPs were observed (Figure 3a), while after 1 min, AuNP aggregates were formed (Figure 3b). After 3 min, short AuNWs were obtained (Figure 3c). Upon increasing the reaction time to 5 min, the AuNW length increased (Figure 3d). After 10 min, the AuNWs increased to a length of a few hundred nanometers, while the average diameter remained unchanged (Figure 2a). By contrast, when Au ions were added to an aqueous solution of HEPES without NaBH_4_ and stirred at 100 °C, only spherical AuNPs with an average diameter of 7.6 nm were prepared (Figure 4a). The UV–vis spectrum exhibited an SP band at 518 nm, confirming the preparation of spherical AuNPs (Figure 4b). Here, Au ions were reduced by HEPES having weak reduction power.^45,50,60^ In addition, the UV–vis spectrum and the TEM image (Figure S4) showed that AuNWs were not prepared when an aqueous solution of NaBH_4_ (24 mM) was added to aqueous solutions of HAuCl_4_·4H_2_O (24 mM) and HEPES (15 mM). Notably, NW synthesis must be conducted under high-concentration conditions of Au nanocrystals to aggregate them (Figure 1). Therefore, when Au ions were reduced in an aqueous solution of HEPES with a low concentration of NaBH_4_ or without NaBH_4_, spherical AuNPs were formed because of the low concentration of Au nanocrystals (Figure 1).

**Figure 5:**
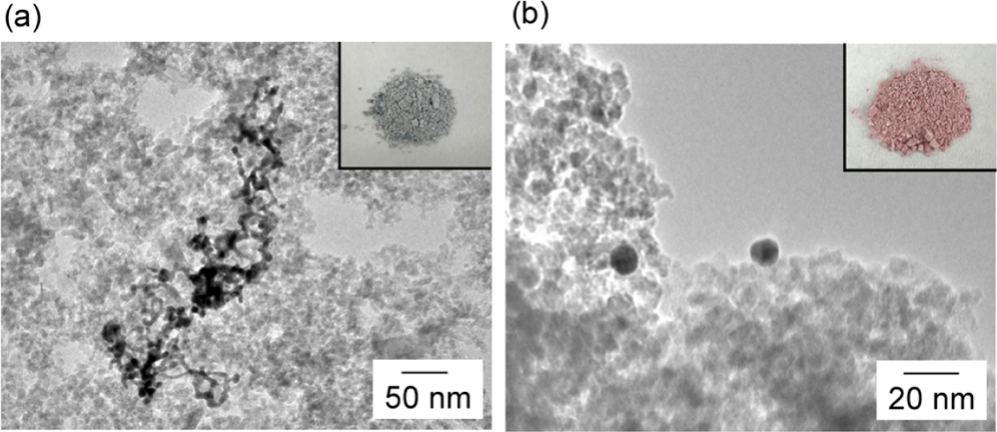
TEM and photographic images of (a) AuNW/γ-Al_2_O_3_ and (b) AuNP/γ-Al_2_O_3_ after water extraction four times.

Before examining the catalytic performance of the synthesized AuNWs, the capping agent must be removed to appropriately evaluate their catalytic properties. However, when the capping agent was removed from the unsupported Au nanocrystals, the Au nanocrystals were easily aggregated and precipitated. Therefore, to inhibit the aggregation and precipitation of Au nanocrystals, we supported AuNWs on γ-Al_2_O_3_ by adding γ-Al_2_O_3_ to the AuNW dispersion prepared using a 15 mM HEPES solution. The NW structure was not changed by this supporting method (Figure S5), and a gray AuNW/γ-Al_2_O_3_ powder was formed. In addition, we removed HEPES using the water extraction method. TEM images showed that the morphology of the NWs did not change even when the extraction process was repeated four times and the gray color was also retained (Figure 5a). Similarly, we removed HEPES from AuNPs supported on γ-Al_2_O_3_ (AuNP/γ-Al_2_O_3_) by extraction with water, and this process was also repeated four times. The morphology and the red color of the powder were not changed by this method (Figure 5b). Fourier transform infrared (FTIR) spectra showed that AuNW/γ-Al_2_O_3_ and AuNP/γ-Al_2_O_3_ after the extraction method did not confirm the peaks of HEPES (Figure S6).

**Figure 6:**
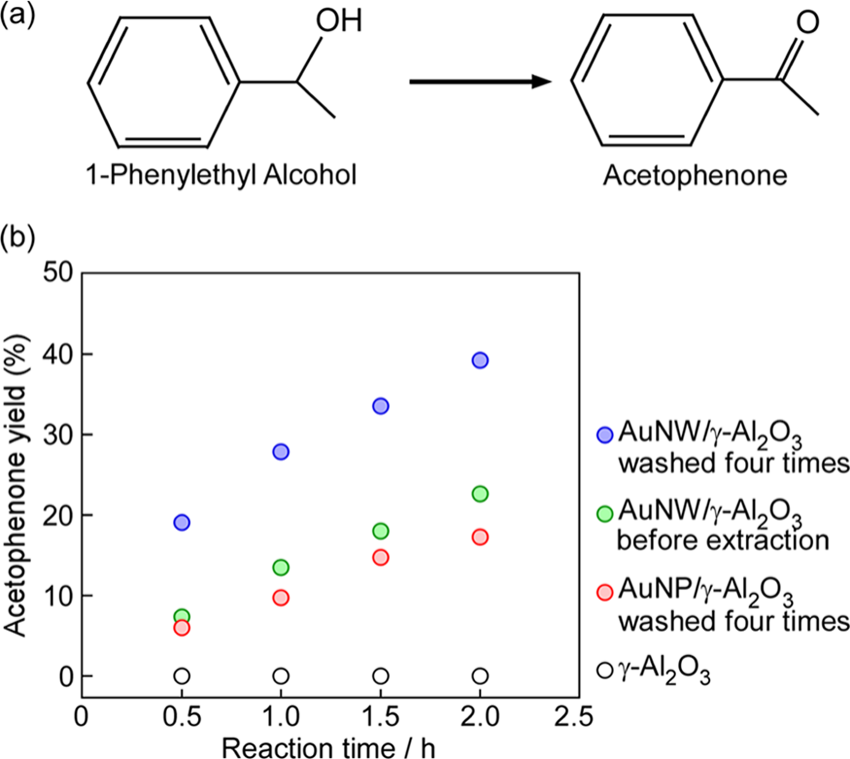
(a) Catalytic oxidation of 1-phenylethyl alcohol. (b) Acetophenone yield using AuNW/γ-Al_2_O_3_ before and after the extraction method, AuNP/γ-Al_2_O_3_ after the extraction method, and γ-Al_2_O_3_. Reaction conditions: 1-phenylethyl alcohol (30 μmol), catalyst (50 mg and Au = 0.63 μmol), K_2_CO_3_ (0.1 g), air (1 atm), and 40 °C.

**Figure 7:**
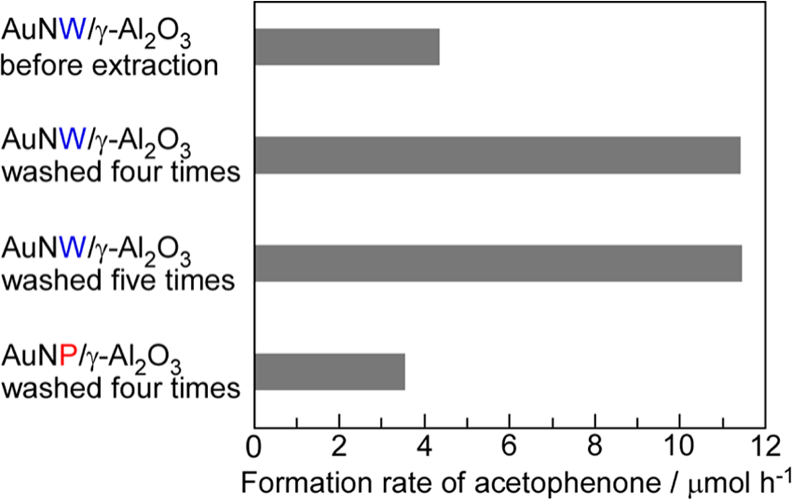
Formation rate of acetophenone after 0.5 h via the oxidation of 1-phenylethyl alcohol. Reaction conditions: 1-phenylethyl alcohol (30 μmol), catalyst (50 mg and Au = 0.63 μmol), K_2_CO_3_ (0.1 g), air (1 atm), 40 °C, and 0.5 h.

We examined the catalytic performance of the catalysts in the oxidation of 1-phenylethyl alcohol to acetophenone using air as an oxidant (Figure 6a). The yield of acetophenone is shown in Figure 6b and Table S1. When γ-Al_2_O_3_ was used without Au nanocrystals, acetophenone was not produced (Figure 6b and Table S1). The formation rate of acetophenone was calculated from the yield of acetophenone after 0.5 h (Figure 7). We confirmed the improvement in catalytic activity after the extraction method by removing HEPES from the Au surface, and AuNW/γ-Al_2_O_3_ washed four times had approximately the same formation rate of acetophenone as AuNW/γ-Al_2_O_3_ when washed five times (Figures 6b and 7). The formation rates over AuNW/γ-Al_2_O_3_ and AuNP/γ-Al_2_O_3_ were 11.3 and 3.5 μmol/h, respectively (Figures 6b and 7). This result shows that the formation rate of acetophenone over AuNW/γ-Al_2_O_3_ was 3.2 times that over AuNP/γ-Al_2_O_3_ (Figure 7). When the NWs and NPs had similar diameters, the surface area of spherical NPs was larger than that of NWs because the NW structure was formed by the aggregation of spherical NPs. These results indicate that the formation rate per Au surface area over AuNW/γ-Al_2_O_3_ was more than 3.2 times that over AuNP/γ-Al_2_O_3_. This NW structure was formed using the aggregation method. The aggregation of nanocrystals increases the catalytic activity compared to the spherical AuNPs having similar diameters because the aggregated domains of the nanocrystals have many disordered metal atoms, namely, grain boundaries. The boundary was confirmed by HRTEM observations (dotted lines in Figure S7). The disordered Au atoms (grain boundary) show a lower coordination number compared to Au atoms on spherical AuNPs because spherical AuNPs were formed by a large number of (111) facets.^1,23^ Additionally, Au atoms with low coordination numbers have high catalytic activity compared to Au atoms with a high coordination number.^61^ Therefore, the formation rate of acetophenone over AuNW/γ-Al_2_O_3_ was 3.2 times that over AuNP/γ-Al_2_O_3_.

## 3 Conclusions

In this study, we prepared AuNWs in an aqueous solution of HEPES, which has a weak affinity to the Au surface, and NaBH_4_ and examined the catalytic performance of the supported AuNWs for the alcohol oxidation of 1-phenylethyl alcohol to acetophenone. The AuNWs had an average diameter of 7.7 nm and a length of a few hundred nanometers. The formation rate of acetophenone over AuNWs supported on γ-Al_2_O_3_ was 3.2 times that over spherical AuNPs supported on γ-Al_2_O_3_.

## 4 Materials and Methods

### 4.1 Materials

Hydrogen tetrachloroaurate tetrahydrate (HAuCl_4_·4H_2_O) was obtained from Nacalai Tesque, Inc. (Japan). NaBH_4_ and γ-Al_2_O_3_ were obtained from Kanto Chemicals (Japan). 1-Phenylethyl alcohol (Tokyo Chemical Co.) and HEPES (Sigma-Aldrich) were used without further purification.

### 4.2 Preparation of AuNW/γ-Al_2_O_3_

An aqueous solution of HAuCl_4_·4H_2_O (24 mM, 200 μL, and 5 μmol) was added to an aqueous solution of HEPES (15, 50, 100, and 200 mM, 10 mL, and pH = 11.9). AuNWs were obtained by adding an aqueous solution of NaBH_4_ (110 mM and 100 μL) to the mixture of HAuCl_4_·4H_2_O and HEPES (10.2 mL) and allowing it to stand for 10 min. AuNW/γ-Al_2_O_3_ was obtained by adding γ-Al_2_O_3_ (0.38 g) to the AuNW dispersion (10.3 mL) prepared using a 15 mM HEPES solution and stirring for 15 h. The AuNW/γ-Al_2_O_3_ powder was collected by centrifugation. HEPES, the capping agent in the collected AuNW/γ-Al_2_O_3_, was removed by extraction with water by adding water to the AuNW/γ-Al_2_O_3_ powder and allowing it to stand for 10 min. AuNW/γ-Al_2_O_3_ was subsequently recovered from the suspension by centrifugation, and this treatment was repeated four times.

### 4.3 Preparation of AuNP/γ-Al_2_O_3_

An aqueous solution of HEPES (75 mM, 500 μL, and pH = 11.9) was added to an aqueous solution of HAuCl_4_·4H_2_O (0.6 mM, 8 mL, and 5 μmol). AuNPs were obtained by stirring the mixture of aqueous solutions for 1 h at 100 °C.

An aqueous solution of HAuCl_4_·4H_2_O (24 mM and 200 μL) was added to an aqueous solution of HEPES (15 mM, 10 mL, pH = 11.9). AuNPs were obtained by adding an aqueous solution of NaBH_4_ (24 mM and 100 μL) to the mixture of HAuCl_4_·4H_2_O and HEPES (10.2 mL) and allowing it to stand for 1 h.

AuNP/γ-Al_2_O_3_ was prepared by adding γ-Al_2_O_3_ to a AuNP dispersion without NaBH_4_ and stirring for 15 h. The AuNP/γ-Al_2_O_3_ powder was collected by centrifugation. HEPES was removed by extraction with water by adding water to the AuNP/γ-Al_2_O_3_ powder and allowing it to stand for 10 min. AuNP/γ-Al_2_O_3_ was subsequently recovered from the suspension by centrifugation, and this treatment was repeated four times.

### 4.4 Catalytic Reaction

The aerobic oxidation of 1-phenylethyl alcohol was conducted in a batch reactor at 40 °C in atmospheric air. After the addition of K_2_CO_3_ (0.1 g and 0.72 mmol) to an aqueous solution of 1-phenylethyl alcohol (3.0 mM, 10 mL, and 30 μmol), the mixture was stirred at 40 °C. AuNW/γ-Al_2_O_3_ and AuNP/γ-Al_2_O_3_ (50 mg) were then added and the mixture was stirred at 40 °C in air (1 atm). The reaction was then quenched with 1 M HCl, and the products were extracted with toluene. The product yield was determined via gas chromatography based on internal standards.

### 4.5 Characterization

TEM was conducted using a JEOL JEM-1011 instrument operating at 100 kV. High-resolution TEM (HRTEM) was performed using a JEOL 2100 instrument equipped with an energy-dispersive X-ray (EDX) spectrometer at 200 kV. UV–vis spectroscopy was conducted using a JASCO V-570 spectrometer. Fourier transform infrared spectroscopy (FTIR) measurements were performed using a Nicolet 6700 FTIR spectrometer equipped with a 4 cm^–1^ resolution. XRD patterns were recorded using a Rigaku Ultima IV diffractometer.

## Supporting Information Available

Molecular structure of HEPES; the TEM-EDX spectrum and the TEM image of AuNWs; the UV–vis spectrum and the TEM image of AuNPs; the TEM image of AuNW/γ-Al_2_O_3_; FTIR spectra of HEPES, AuNW/γ-Al_2_O_3_, AuNP/γ-Al_2_O_3_, and γ-Al_2_O_3_; catalytic performance of γ-Al_2_O_3_, AuNW/γ-Al_2_O_3_, and AuNP/γ-Al_2_O_3_; and the HRTEM image of AuNW (PDF)
