# Supplementary material for: Preparing Alumina-Supported Gold Nanowires for Alcohol Oxidation
Source: ACS Omega. 2021 Jun 13;6(24):16043–8. doi: 10.1021/acsomega.1c01895 (PMC8223421; doi:10.1021/acsomega.1c01895)
Supplement: Supplementary file 1 — ao1c01895_si_001.pdf [file ao1c01895_si_001.pdf]

Preparing Alumina-Supported Gold Nanowires for Alcohol Oxidation

Yoshiro Imura,[1,*] Motoki Maniwa,[1] Kazuki Iida,[1] Haruna Saito,[1] Clara Morita-Imura,[2] and Takeshi Kawai[1,*]

[1]Department of Industrial Chemistry, Tokyo University of Science, 1-3 Kagurazaka, Shinjuku-ku, Tokyo 162-8601, Japan.

[2]Department of Chemistry, Faculty of Science, Ochanomizu University, 2-1-1 Otsuka, Bunkyo-ku, Tokyo 112-8610, Japan.

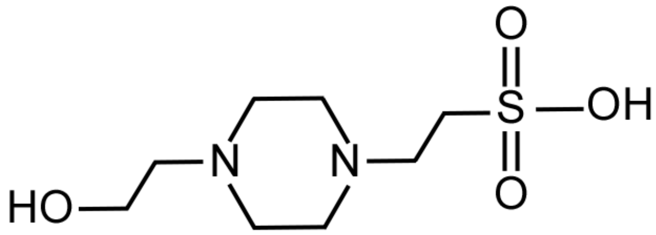

Figure S1. Molecular structure of HEPES.

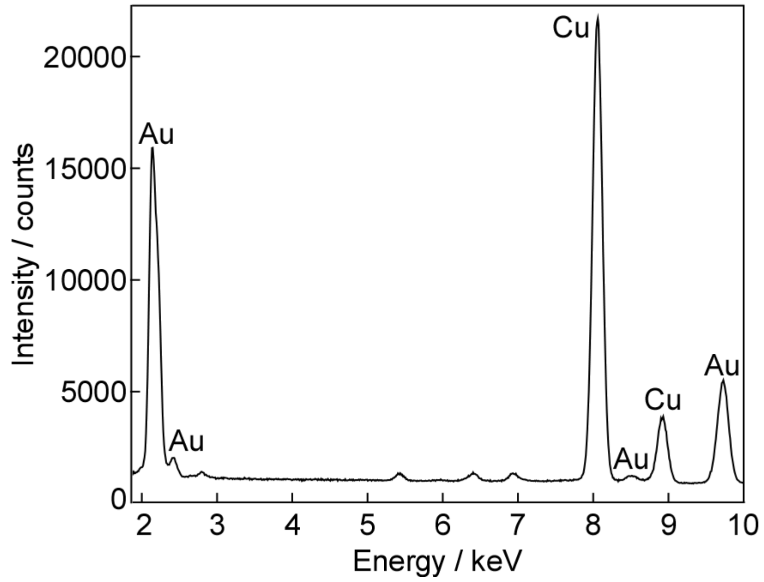

Figure S2. TEM-EDX spectrum of AuNWs. The Cu peaks arise from the TEM Cu grid.

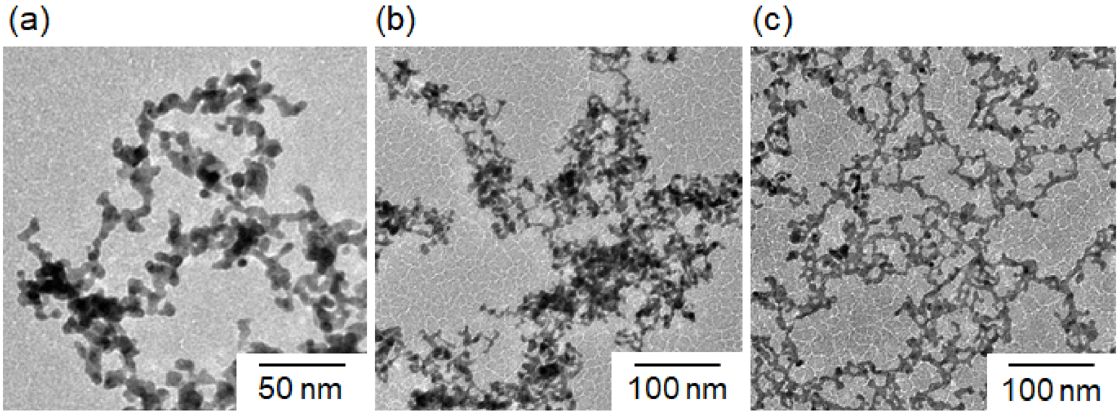

Figure S3. TEM images of AuNWs prepared using (a) 50, (b) 100, and (c) 200 mM HEPES aqueous solution.

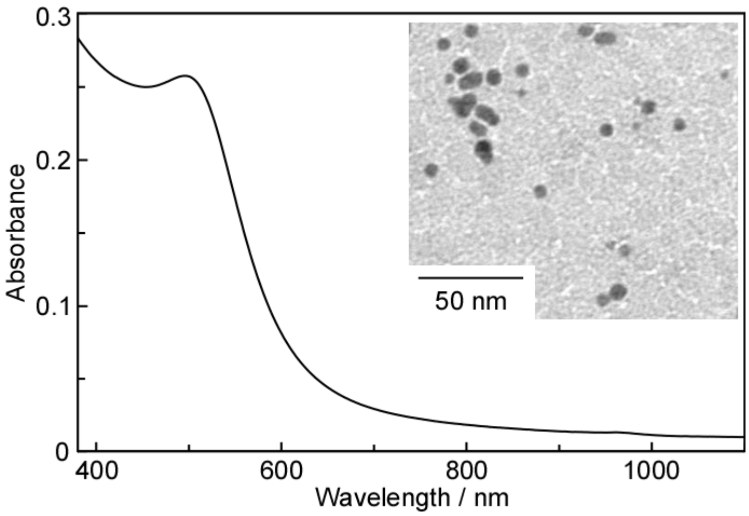

Figure S4. UV-vis spectrum and TEM image of Au nanocrystals. The concentrations of HEPES and $NaBH_4$ are 15 and 24 mM, respectively.

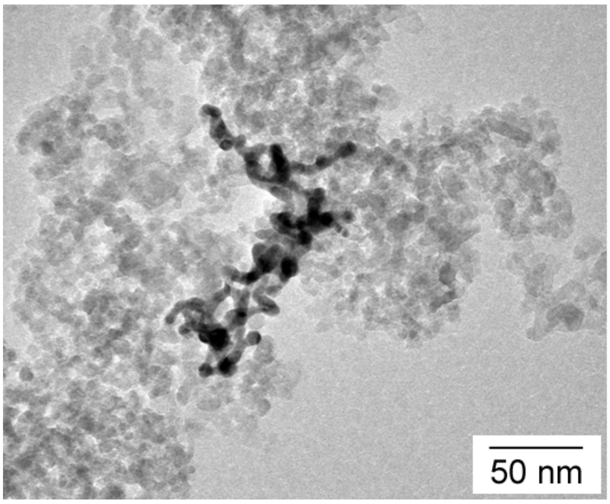

Figure S5. TEM image of AuNW/γ-Al_2O_3 before water extraction.

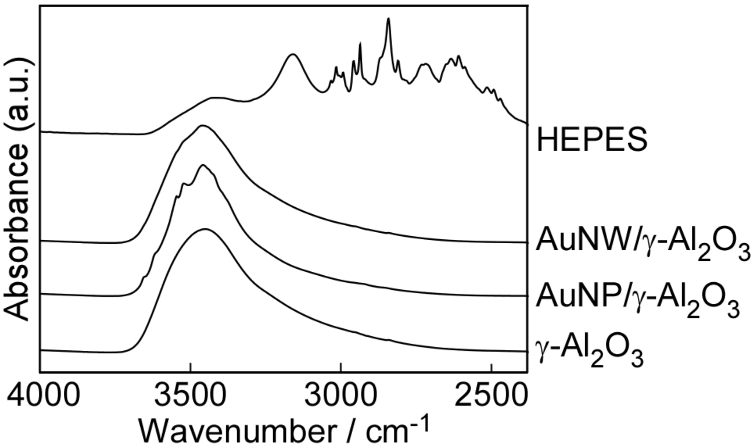

Figure S6. FT-IR spectra of HEPES, AuNW/γ-Al_2O_3, AuNP/γ-Al_2O_3, and γ-Al_2O_3.

Table S1. Catalytic performance of γ-Al_2O_3, AuNW/γ-Al_2O_3, and AuNP/γ-Al_2O_3.

	1-PA conversion (%)	AP yield (%)
γ-Al_2O_3	0	0
AuNW/γ-Al_2O_3 after water extraction four times	41	39
AuNP/γ-Al_2O_3 after water extraction four times	18	17

1-PA: 1-phenylethyl alcohol, AP: acetophenone

Reaction conditions: 1-phenylethyl alcohol (30 μmol), catalyst (50 mg and Au = 0.63 μmol), K_2CO_3 (0.1 g), air (1 atm), 40 °C and 2 h.

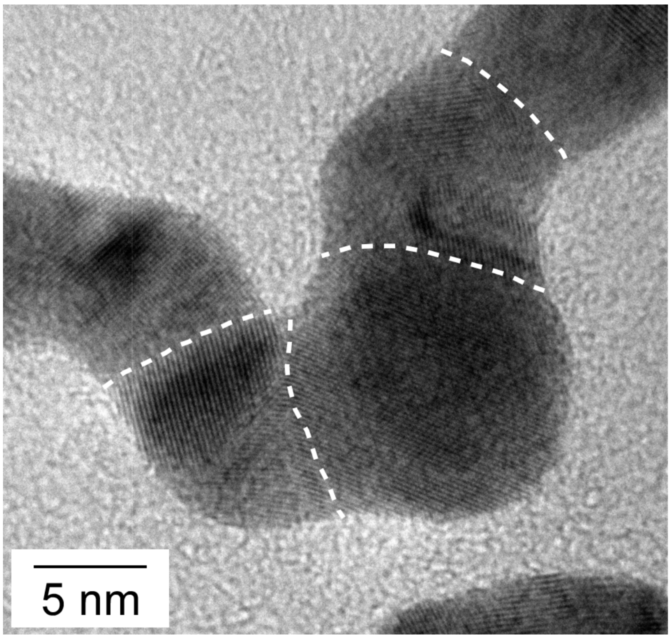

Figure S7. HR-TEM image of AuNW.
